# Induction of CD4 T cell memory responses following BCG vaccination in cattle

**DOI:** 10.3389/fvets.2024.1491424

**Published:** 2024-11-27

**Authors:** Haley M. Sterle, Ellie J. Putz, Steven C. Olsen, Paola M. Boggiatto

**Affiliations:** ^1^National Animal Disease Center, Agricultural Research Service (USDA), Ames, IA, United States; ^2^Iowa State University Immunobiology Graduate Program, Ames, IA, United States

**Keywords:** BCG, cattle, CD4, T helper 1, immunological memory

## Abstract

*Mycobacterium bovis*, the causative agent of bovine tuberculosis (bTB), is a zoonotic pathogen that contributes to economic losses in the cattle industry and poses a public health risk worldwide. Bacillus Calmette-Guerin, or BCG, is a live attenuated strain of *M. bovis* that is used for human vaccination against tuberculosis and is considered a potential vaccine candidate against bTB. However, BCG affords widely variable levels of protection against challenge and interferes with current diagnostic methods, and as such, it is not currently approved for use as a livestock or wildlife vaccine in the United States. Many efforts have been made to develop bTB vaccines that are reliable and do not interfere with diagnostic testing, but BCG continues to be the most effective option. Previous work has shown that a T helper 1 immune response is essential for protection against virulent *M. bovis* infection, characterized by CD4^+^ central and effector memory T cells. In an effort to identify an efficacious bTB intervention strategy, the study presented here used an *in vitro* recall response assay and concurrent evaluation of CD4^+^ T cell proliferation and cytokine production to characterize the surface and functional phenotypes of memory responses to BCG vaccination in cattle. Our findings enhance understanding of the bovine immune response to BCG and provide insights into the development of improved vaccines for the control of bTB.

## 1 Introduction

Bacillus Calmette-Guerin (BCG) is a live attenuated strain of *Mycobacterium bovis*, the causative agent of bovine tuberculosis (bTB), that was developed for human vaccination against *Mycobacterium tuberculosis* in the early 20^th^ century (1). BCG is still administered to infants in developing countries where tuberculosis (TB) is prevalent in humans but is not used in the United States. BCG is also known to be protective against bTB, though efficacy is variable, and vaccination interferes with diagnostics used for cattle and other wildlife reservoirs (1). Therefore, BCG is not licensed for use in livestock or wildlife in the United States. Though efforts have been made to develop reliable vaccines against TB and bTB that do not interfere with diagnostic skin testing (2, 3), BCG remains the most efficacious option to prevent disease.

*M. bovis* is a zoonotic, intracellular pathogen that primarily infects macrophages (4, 5) and results in granulomatous lesions of the lung and lymph nodes (6). CD4^+^ T cells and interferon gamma (IFN-γ) production are instrumental in controlling mycobacterial infections (7, 8), both of which are hallmark characteristics of a T helper 1 (Th1) response (9). Though IFN-γ has been shown to increase mycobacteriostatic activity of macrophages (7), increased levels of IFN-γ are not directly correlated with protection against bTB (10). More recently, *M. bovis* specific CD4^+^ T cells were observed in the peripheral blood of challenged cattle using an *in vitro* recall response assay in conjunction with flow cytometry (11), allowing for the identification of individual functional populations based on concurrent evaluation of proliferative capability and cytokine production of T cell subsets.

Memory T cells induced by vaccination are essential for protection against infections. In cattle, activated memory cells express the CD45RO isoform of CD45, whereas naïve and effector T cells express CD45RA and are CD45RO^−^ (12). Activated memory T cells (i.e., CD45RO^+^ T cells) consist of a heterogeneous population of cells including central memory (T_CM_), effector memory (T_EM_), and resident memory (T_RM_) T cells; each characterized by distinct expression of surface molecules, function, and migration patterns (13). T_CM_ and T_EM_ populations are both found in peripheral blood (14) and are differentiated by their unique proliferative capacities, effector functions, and expression of surface markers CD62L and CCR7. CD62L, or L-selectin, mediates rolling adhesion of T cells along high endothelial venules (HEVs). CCR7 is a chemokine receptor for CCL19 and CCL21, which are found in high concentrations on HEVs and allow firm attachment to endothelial cells for extravasation of T cells into lymph nodes (15, 16). T_CM_, defined as CD45RO^+^ CCR7^+^ CD62L^+/high^ (16), circulate through secondary lymphatic tissues (SLT), proliferate rapidly in response to cognate antigen recognition, and can differentiate into effector T cells and T_EM_ cells (14, 17). T_EM_ are defined as CD45RO^+^ CCR7^−^ CD62L^−/low^ (16), have a low proliferative potential, exhibit strong effector functions, and remain in the periphery (14). Unlike T_CM_ and T_EM_, T_RM_ are preferentially located in peripheral tissues, and do not recirculate (18).

Previously, CD4^+^ T cells exhibiting surface phenotypes consistent with T_CM_ and T_EM_ responses were identified in cattle infected with *M. bovis* (17). Functional characteristics of CD4^+^ T cells, specifically proliferation and IFN-γ production, were measured independently in PBMC cultures stimulated with mycobacterial antigens and supplemented with exogenous IL-2. IFN-γ or proliferating CD4^+^ T cells were further characterized into effector (CD45RO^−^ CCR7^−^), T_CM_ (CD45RO^+^ CCR7^+^) or T_EM_ (CD45RO^+^ CCR7^−^) populations. After extended *in vitro* stimulation with mycobacterial antigens, a majority of IFN-γ^+^ CD4^+^ T cells exhibited a T_CM_ phenotype, while a T_EM_ phenotype was predominant in proliferating CD4^+^ T cells. Further understanding of the functional potential of CD4^+^ T_CM_ and T_EM_ from *M. bovis* infected cattle was prevented, as the functional parameters were measured independently rather than concurrently.

This study was conducted with the goal of further characterizing long-term memory CD4^+^ T cell responses induced by BCG vaccination of cattle. Briefly, we utilized an *in vitro* recall response assay to concurrently evaluate proliferation, IFN-γ production, and surface marker expression of CD4^+^ T cells to thoroughly describe the phenotypic and functional heterogeneity of memory cells following BCG vaccination. Our data will allow for comparison of functional T cell responses to vaccination and aid in identification of an efficacious bTB vaccine.

## 2 Materials and methods

### 2.1 Vaccination

Yearling Angus cross heifers (*n* = 8) were housed in an outdoor facility at the National Animal Disease Center (NADC) in Ames, Iowa. All heifers were vaccinated subcutaneously with 1 mL containing 2.4 × 10^5^ colony forming units of BCG Danish 1331. Routine protocols to maintain the health and wellbeing of animals during the study were implemented. All animal procedures received prior approval from the NADC Animal Care and Use committee (ARS-21-0990, approval date 02/11/2022).

### 2.2 Peripheral blood mononuclear cell (PBMC) isolation

Whole blood was collected from all heifers at the time of vaccination and at four-week intervals until 24 weeks post-vaccination to assess peripheral immune responses. Thirty mL of blood were collected via venipuncture of the jugular vein and added to 3 mL of acid citrate dextrose (ACD) anticoagulant. PBMCs were isolated as previously described (19). Live cell count was determined for each sample using the Muse® Count and Viability Kit on the Guava® Muse® Cell Analyzer (Luminex). Cell suspensions were adjusted to a final concentration of 1×10^7^ cells per mL in complete 1640 RPMI (cRPMI) media containing 20% heat-inactivated FBS, 1% HEPES, 1% non-essential amino acids, 1% essential amino acids, 1% sodium pyruvate, 100 U/mL penicillin, 100 μg/mL streptomycin, 2 nM glutamine, and 50 μM 2-beta mercaptoethanol.

### 2.3 Labeling PBMCs for proliferation assay

PBMCs were labeled with the CellTrace™ Violet (CTV) proliferation kit (Cat. No. C34557, Thermo Fisher Scientific) to track *in vitro* proliferation. PBMC labeling was conducted according to the manufacturer’s recommendations with minor modifications. Briefly, CTV dye was reconstituted in 20 μL dimethyl sulfoxide (DMSO) provided by the manufacturer, suspended in 780 μL PBS, and further diluted 1:10 in sterile Dulbecco’s phosphate buffered saline (DPBS). PBMCs were washed in DPBS and centrifuged at 300x g for 10 min at room temperature (RT). Supernatants were discarded and cell pellets were resuspended in diluted CTV, then vortexed and incubated for 20 min at RT with occasional vortexing. Cells were then washed in DPBS and again centrifuged at 300x g for 10 min at RT. Supernatants were discarded, and PBMC were resuspended to a final concentration of 1×10^7^ cells per mL in cRPMI.

### 2.4 *In vitro* recall response assay

To assess *in vitro* recall responses, 100 μL of CTV-labeled cRPMI PBMC suspensions (1×10^6^ cells) were plated per well in 96 well flat bottom plates and treated with various stimulation conditions. PBMCs were then left unstimulated (media only) or stimulated with γ-irradiated *Brucella abortus* strain RB51 antigen (10^7^ CFU/well), purified protein derivative of *Mycobacterium bovis* (PPDb), or Concanavalin A (ConA). All stimulation conditions were plated in duplicate. Plates were incubated for 7 days at 37°C with 5% CO_2_. Sixteen hours prior to beginning cell staining procedure on day 7, all cells were either treated with eBioscience™ protein transport inhibitor (Cat. No. 00–4980-93, Thermo Fisher Scientific) or restimulated with eBioscience™ cell stimulation cocktail plus protein transport inhibitors (Cat. No. 00–4975-93, Thermo Fisher Scientific) for assessment of intracellular cytokine production.

### 2.5 Surface marker and intracellular cytokine staining

PBMCs were harvested on day 7 and washed twice in DPBS at 300x g for 5 min at RT. PBMCs were then incubated with eBioscience eFluor™ 780 fixable viability dye (Cat. No. 65–0865-14, Thermo Fisher Scientific) for 20 min at 4°C and subsequently washed via centrifugation as previously described, once with DPBS and once with FACS buffer (PBS + 0.5% fetal bovine serum (FBS)). PBMCs were incubated with a primary anti-bovine CD45RO antibody (clone IL-A116, Cat. No. MCA2434GA, BioRad) for 15 min at room temperature and then incubated with a secondary anti-mouse IgG3 BUV395-labeled antibody (clone R40-82, Cat. No. 744138, BD Biosciences) for 15 min at RT. FACS buffer was used to wash cells twice, and cells were then incubated with FITC labeled anti-bovine CD4 (clone CC8, Cat. No. MCA1653F, BioRad), BV650 labeled anti-human CD62L (clone DREG-56, Cat. No. 304832, BioLegend), and PE-Cy7 labeled anti-human CCR7 (clone 3D12, Cat. No. 557648, BD Biosciences) antibodies for 15 min at RT. Following incubation and two washes in FACS buffer, cells were fixed and permeabilized using the BD Cytofix/Cytoperm kit (Cat. No. 554714, BD Biosciences) according to manufacturer’s recommendations. Intracellular staining was then carried out by incubating cells with PE labeled anti-bovine IFN-γ antibody (clone CC302, Cat. No. MCA1783PE, BioRad) for 30 min at room temperature. Cells were washed once with 1X wash/perm buffer and once with FACS buffer. Cells were then resuspended in 200 μL FACS buffer and proliferation, cytokine production, and surface markers were concurrently analyzed using a BD FACSymphony A5 flow cytometer (BD Biosciences). A total of 50,000 live cells were targeted for collection. Additionally, all data analyzed used a threshold of at least 1,000 live CD4^+^ T cells for inclusion in the analysis. Resulting data was analyzed using FlowJo software (version 10.8).

### 2.6 Data and statistical analysis

The study was designed to follow vaccine-induced T cell responses over 24 weeks, comparing functional and phenotypic results back to control samples at the Day 0 pre-vaccination time point. Internal assay controls to evaluate the effect of environmental factors and potential non-cognate antigen responses were also included at each time point with unstimulated, RB51 stimulated, and ConA stimulated PBMCs.

All data were analyzed using a simple auto regressive model (AR1) in R (version 4.2.0). Time point (weeks post vaccination) was set as a fixed effect for all data. Depending on the data, either stimulation condition or cell type and the interaction between weeks post vaccination were also set as fixed effects. Pairwise comparisons of Least Squares Means were conducted to determine significant differences between specific contrasts of interest such as stimulation conditions or cell types at each time point. Significance was determined when *p*-value ≤0.05, and error bars represent standard errors.

## 3 Results

### 3.1 CD4^+^ T cell proliferative responses following BCG vaccination

**Figure 1:**
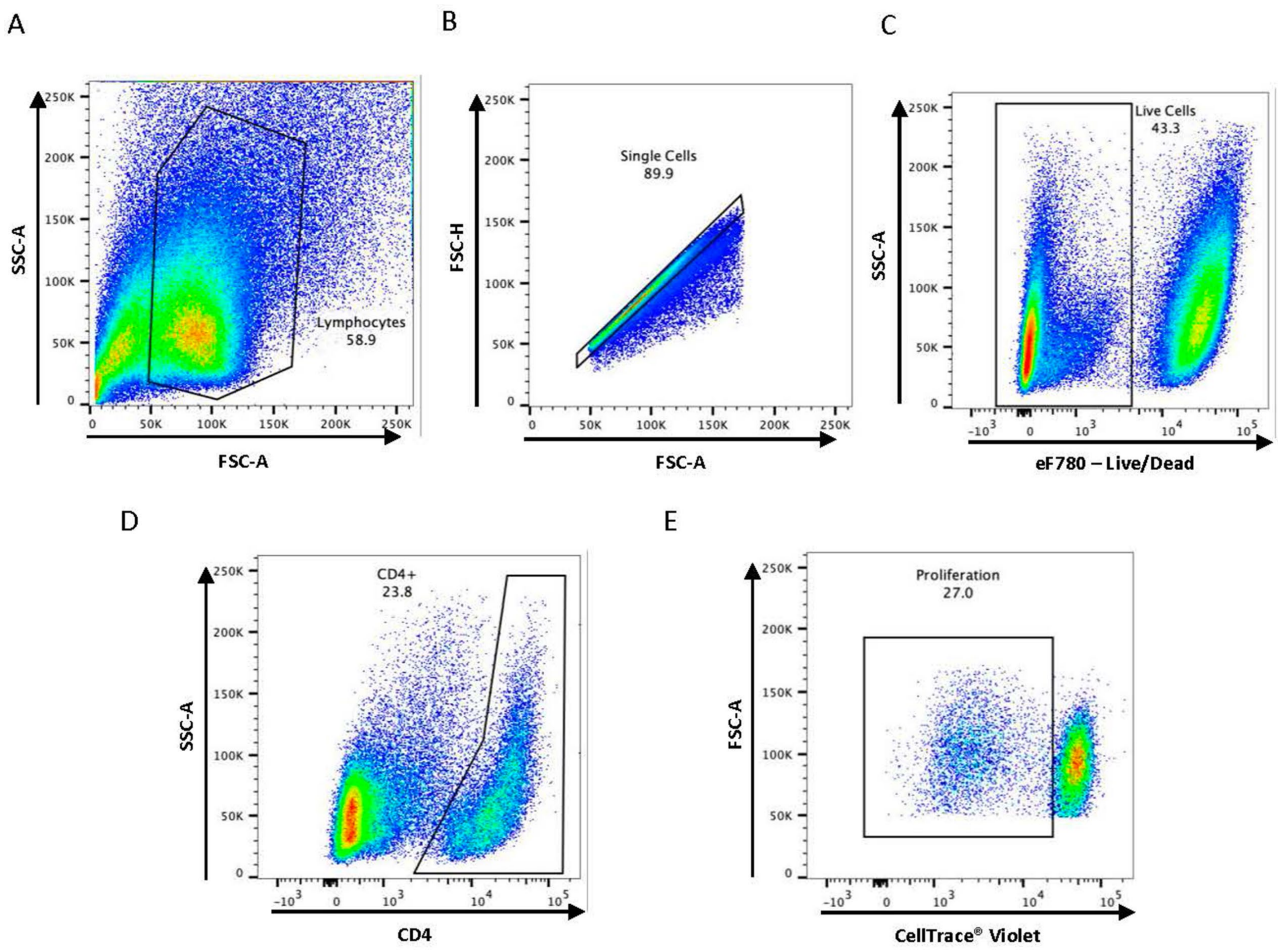
Gating strategy used for flow cytometry analysis. Representative dot plots are shown describing the gating strategy for **(A)** lymphocytes, **(B)** single cells, **(C)** live cells, **(D)** CD4^+^ T cell subset, and **(E)** antigen-specific proliferation.

**Figure 2:**
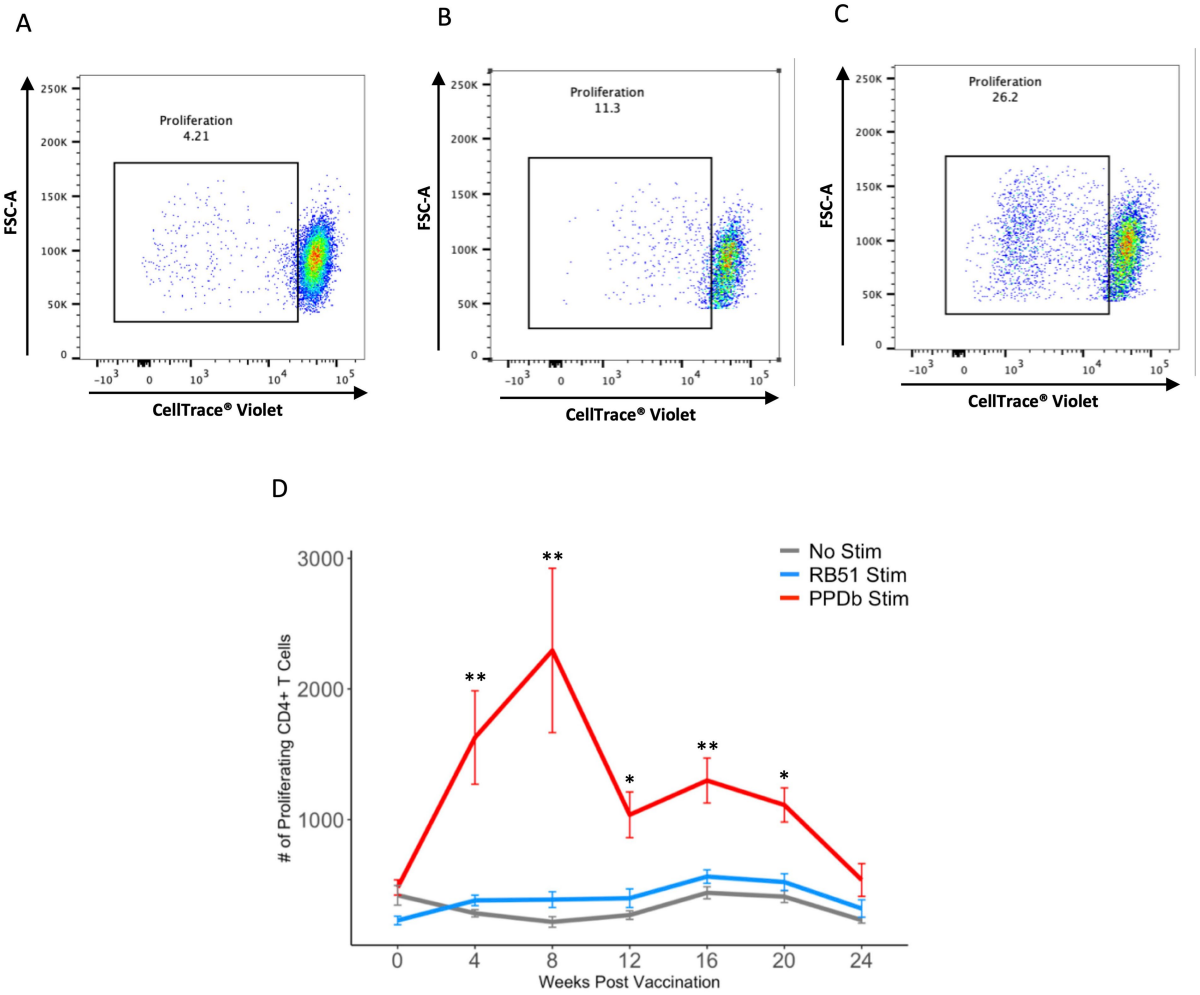
PPDb-specific CD4^+^ T cells proliferate in response to stimulation. Representative dot plots from one animal showing proliferation of CD4^+^ T cells at 8 weeks post vaccination in response to **(A)** media only (unstimulated cells), **(B)** γ-irradiated RB51, and **(C)** PPDb. **(D)** Mean numbers of proliferating CD4^+^ T cells following 7 days of either *in vitro* PPDb stimulation (red line), *in vitro* RB51 stimulation (blue line), or unstimulated cells (gray line). Error bars represent standard errors. ** p ≤ 0.01, *** p ≤ 0.001.

We first characterized the population of CD4^+^ T cells responding to BCG vaccination in the peripheral blood of vaccinated animals. PBMCs were stimulated *in vitro* under various conditions and flow cytometry was used to identify BCG-specific CD4^+^ T cells using proliferation as a marker of antigen specificity (Figure 1). When PBMCs from BCG vaccinated cattle were left unstimulated or stimulated with an unrelated antigen (γ-irradiated RB51), a CD4^+^ T cell proliferative response was not observed at any of the time points analyzed (Figure 2). Conversely, when PBMCs were stimulated with PPDb, a significant increase in the number of proliferating CD4^+^ T cells was observed as compared to unstimulated controls (*p* < 0.01) or RB51 stimulated CD4^+^ T cells (*p* < 0.05, not shown) at 4, 8, 12, 16, and 20 weeks post vaccination (Figure 2). Peak proliferative CD4^+^ T cell responses were observed at 8 weeks post vaccination which subsequently declined until the end of the study. Our data demonstrated that BCG vaccination elicited a significant antigen specific CD4^+^ T cell response in the peripheral blood of cattle.

### 3.2 CD45RO expression within the PPDb-specific CD4^+^ T cell population

**Figure 3:**
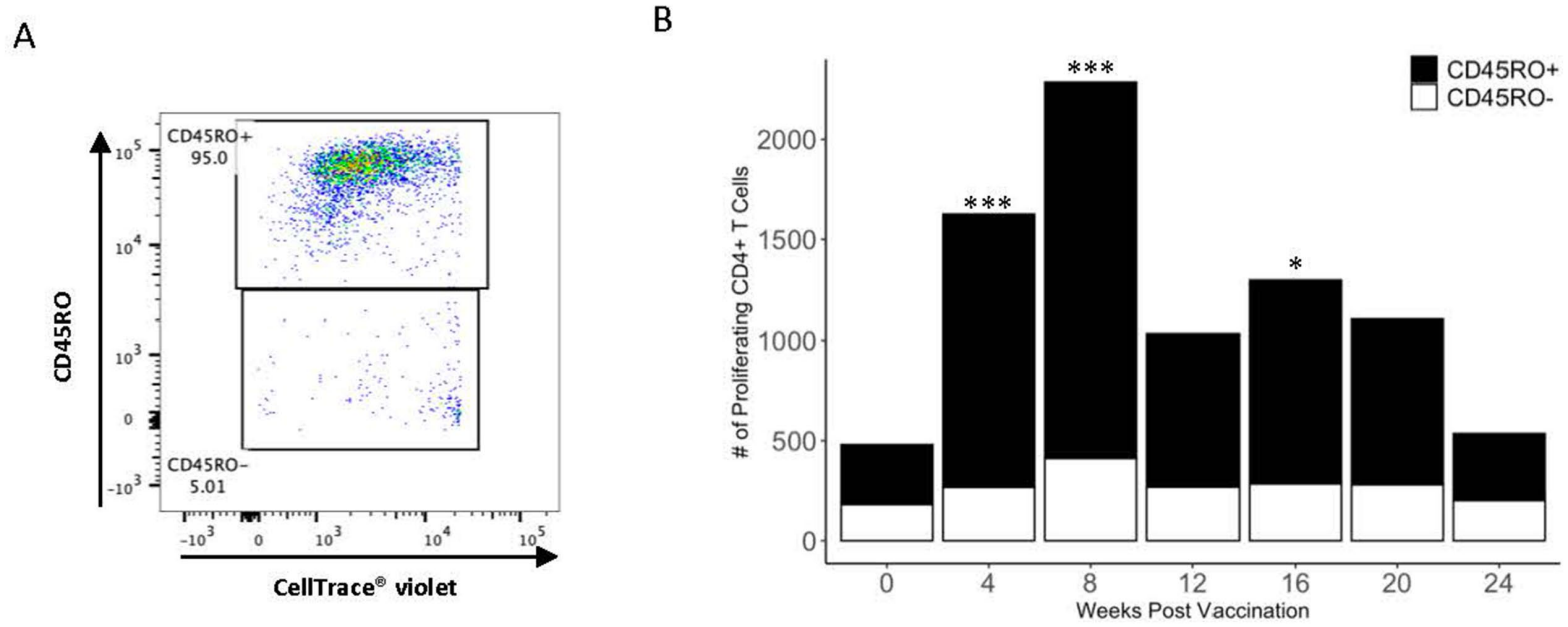
PPDb-specific CD4^+^ T cells preferentially exhibit the CD45RO^+^ phenotype. A representative dot plot shows discrimination of PPDb-specific CD45RO^+^ and CD45RO^−^ CD4^+^ T cells during flow cytometry gating **(A)**. The number of PPDb-specific CD45RO^+^ (black bars) is compared to PPDb-specific CD45RO^−^ (white bars) at each time point following vaccination with BCG. CD45RO^+^ and CD45RO^−^ bars together represent total number of proliferating CD4^+^ T cells in response to 7 days of *in vitro* PPDb stimulation at each time point **(B)**. * p ≤ 0.05, *** p ≤ 0.001.

After establishing the presence of a PPDb-specific CD4^+^ T cell population, CD45RO surface expression was analyzed to differentiate activated memory cells from effector cells. Proliferating CD4^+^ T cells were gated as either CD45RO^+^ or CD45RO^−^ (Figure 3A). As shown in Figure 3B, the majority (> 70%) of the PPDb-specific CD4^+^ T cells were CD45RO^+^ at 4, 8, 12, 16, and 20 weeks post vaccination. Additionally, the number of PPDb-specific activated memory CD4^+^ T cells significantly increased at 4, 8, and 16 weeks following BCG vaccination when compared to time point 0 (*p* < 0.05) (Figure 3B). Though fewer in number, PPDb-specific CD4^+^ CD45RO^−^ effector T cells were observed at all time points but did not increase significantly at any time point (*p* > 0.40). Ultimately, increases in the number of PPDb-specific CD4^+^ T cells following BCG vaccination were predominantly driven by activated memory CD45RO^+^ CD4^+^ T cells rather than effector CD45RO^−^ CD4^+^ T cells.

### 3.3 Subsets of PPDb-specific memory CD4^+^ T cells

**Figure 4:**
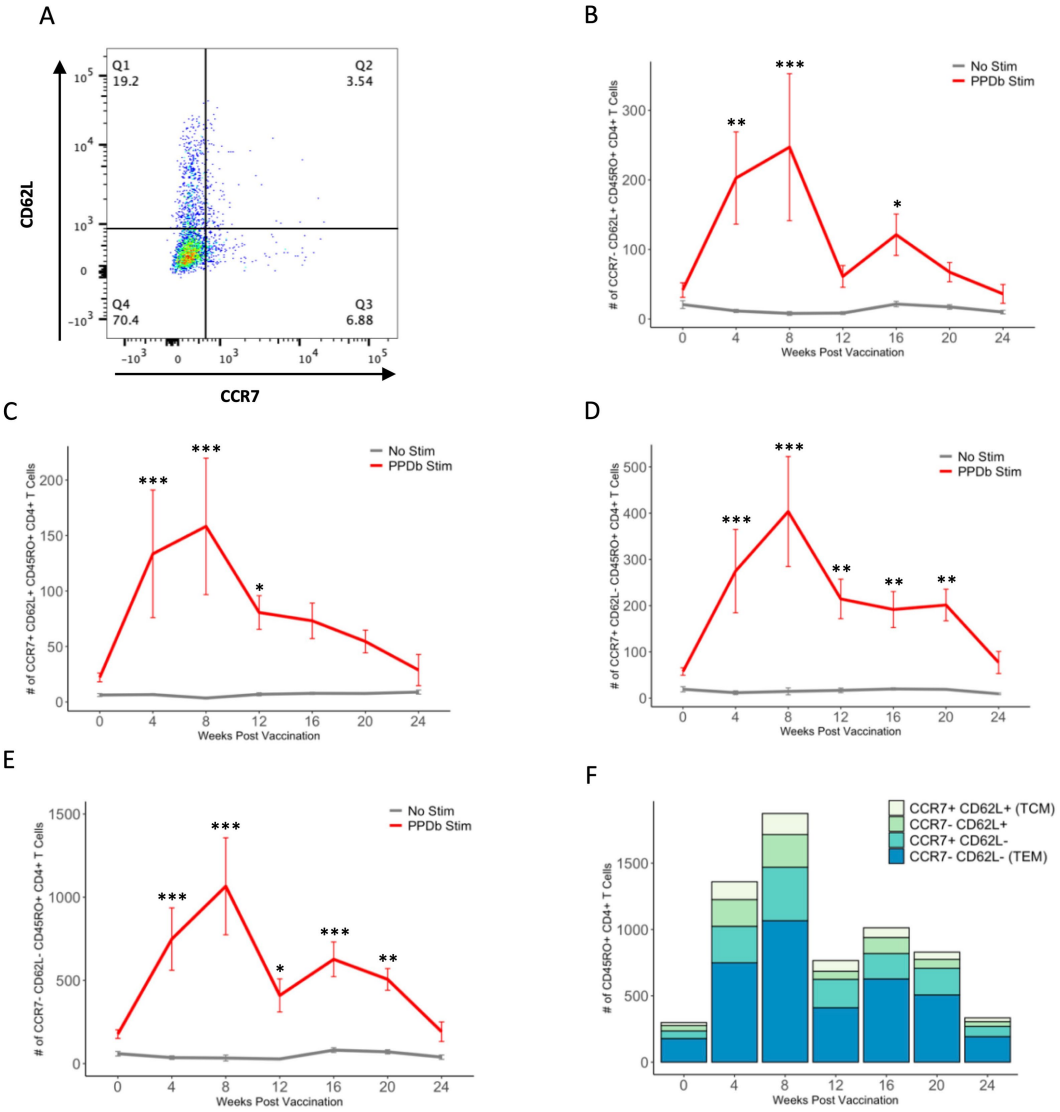
Four subsets of PPDb-specific CD45RO^+^ CD4^+^ T cells are observed following vaccination with BCG. Representative dot plot shows four CD45RO^+^ memory subsets defined by CCR7 and CD62L expression **(A)**. A significant number of CCR7^−^ CD62L^+^ **(B)**, CCR7^+^ CD62L^+^ T_CM_ **(C)**, CCR7^+^ CD62L^−^ **(D)**, and CCR7^−^ CD62L^−^ T_EM_ **(E)** cells are observed after 7 days of *in vitro* PPDb stimulation at various time points when compared to unstimulated cells incubated in media only. Counts of each subset are shown using a standard axis, with the total of all four subsets being equivalent to the overall number of PPDb-specific CD45RO^+^ CD4^+^ T cells at each time point post vaccination **(F)**. *p ≤ 0.05, **p ≤ 0.01, ***p ≤ 0.001.

To further analyze T cell memory subsets elicited following BCG vaccination, PPDb-specific (i.e., proliferating) CD45RO^+^ CD4^+^ T cells were gated based on their surface expression of CCR7 and CD62L. Four populations of cells were identified: CCR7^−^ CD62L^+^, CCR7^+^ CD62L^+^ (T_CM_), CCR7^+^ CD62L^−^, and CCR7^−^ CD62L^−^ (T_EM_) (Figure 4A). We observed a significant increase in the number of cells within all subsets after vaccination. Peak cell numbers were observed at 8 weeks post BCG vaccination, and subsequently declined to pre-vaccination levels by 24 weeks post vaccination (Figures 4B–E). Numbers of CD62L single positive and CCR7/CD62L double negative CD45RO^+^ CD4^+^ T cells were greater (*p* < 0.05) after PPDb stimulation than in unstimulated cultures at 4, 8, 16, and 4, 8, and 12 weeks post vaccination, respectively (Figures 4B–C). However, numbers of CCR7 single positive and CCR7/CD62L double negative CD45RO^+^ CD4^+^ T cells were both greater (*p* < 0.05) in PPDb stimulated cells at 4, 8, 12, 16, and 20 weeks post vaccination when compared to cells in unstimulated cultures (Figures 4D–E). When comparing all populations, the number of cells expressing a T_EM_ phenotype (CCR7/CD62L double negative) were consistently the most prevalent, making up 53–62% of the total number of CD45RO^+^ CD4^+^ T cells at each time point. T_EM_ CD4^+^ T cells were significantly higher at 4, 8, 16, and 20 weeks post vaccination (*p* < 0.05) when compared to the three other subsets. At 12 weeks post vaccination, the number of T_EM_ CD4^+^ T cells were greater (*p* < 0.05) than all other subsets except the CCR7 single positive population (*p* > 0.1) (Figure 4F). At all sampling times, T_CM_ (CCR7/CD62L double positive) cells were the least prevalent memory cell subset, with single positive populations expressed at intermediate levels (Figure 4F). Based on CCR7 and CD62L surface expression, our data indicate the presence of four unique PPDb-specific memory CD4^+^ T cell subsets following BCG vaccination. Furthermore, our data demonstrates that the majority of the CD4^+^ T cell memory population is composed of CCR7^−^ CD62L^−^ CD45RO^+^ CD4^+^ T cells consistent with a T_EM_ phenotype.

### 3.4 IFN-γ production by PPDb-specific CD4^+^ T cell memory subsets

**Figure 5:**
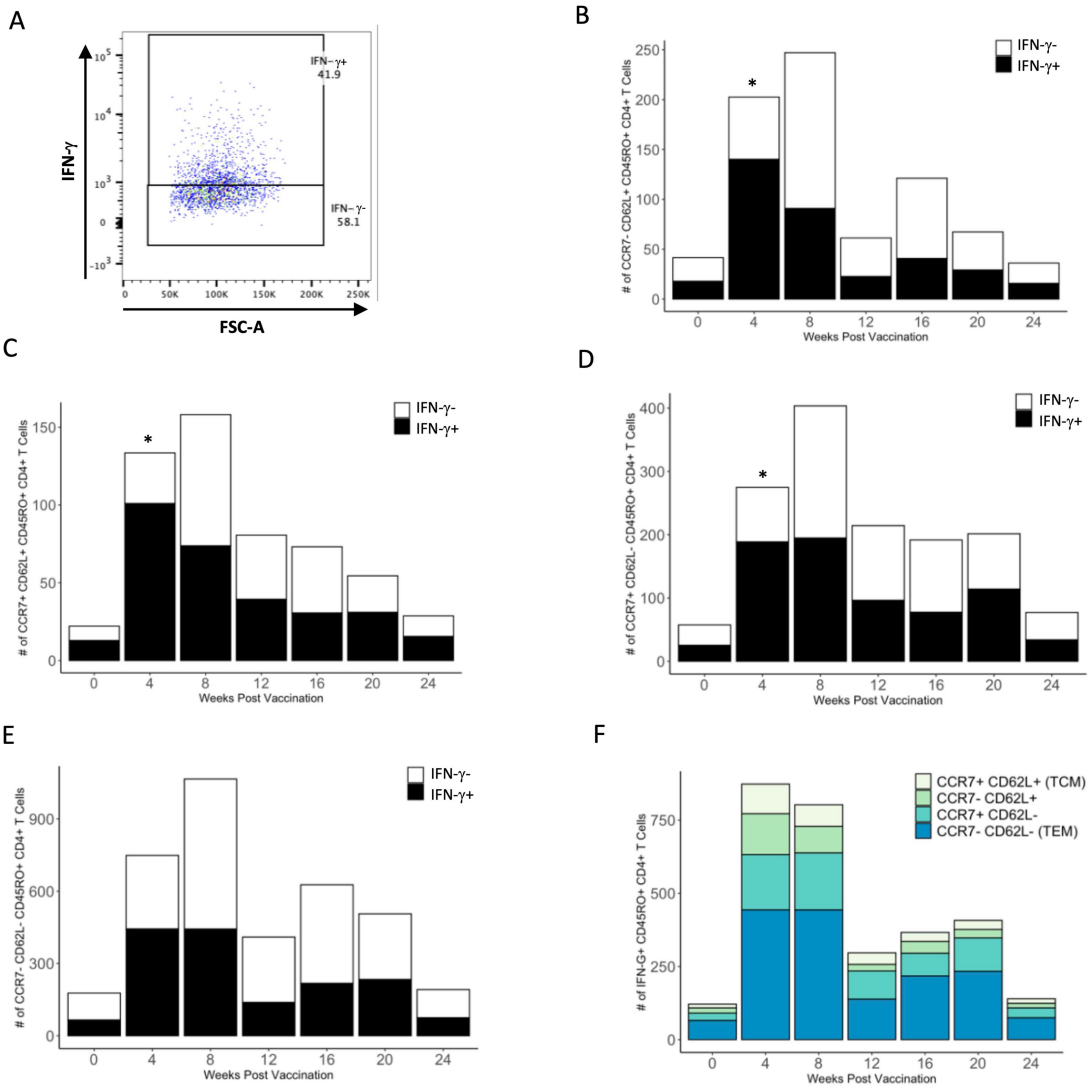
PPDb-specific cells of each CD45RO^+^ subtype produce IFN-γ in response to 7 days of *in vitro* stimulation. Representative dot plot shows gating for PPDb-specific IFN-γ^+^ and IFN-γ^−^ CD45RO^+^ CD4^+^ T cell subsets. Gating is based on IFN-γ single stain control **(A)**. Number of PPDb-specific IFN-γ^+^ (black bars) and IFN-γ^−^ (white bars) CD45RO^+^ CD4^+^ T cells within the CCR7^−^ CD62L^+^ **(B)**, CCR7^+^ CD62L^+^ T_CM_ **(C)**, CCR7^+^ CD62L^−^ **(D)**, and CCR7^−^ CD62L^−^ T_EM_ **(E)** subsets are shown. Counts of IFN-γ^+^ and IFN-γ^−^ cells together represent the total number of unique CD45RO^+^ subset at each time point post vaccination **(B–E)**. Counts of IFN-γ^+^ cells within each subset are shown using a standard axis, with the total count of all four subsets being equivalent to the overall number of PPDb-specific IFN-γ^+^ CD45RO^+^ CD4^+^ T cells at each time point post vaccination **(F)**. *p ≤ 0.05.

We analyzed each PPDb-specific memory subset for IFN-γ production as a measure of effector function in response to antigen stimulation. IFN-γ^+^ and IFN-γ^−^ populations within the four CD4^+^ T cell memory subsets were identified using flow cytometry as shown in Figure 5A. Within all four memory populations, we observed a trend for increased numbers of PPDb-specific IFN-γ^+^ cells following BCG vaccination (Figures 5B–E). CD62L single positive, CD62L/CCR7 double positive, and CCR7 single positive subsets had a significant number of IFN-γ^+^ cells at 4 weeks post vaccination (*p* < 0.05), though the total number of PPDb-specific cells within each subset did not peak until 8 weeks post vaccination. When comparing the number of IFN-γ-producing cells among all subsets, T_EM_ CD4^+^ T cells had greater number of IFN-γ^+^ cells at 4, 8, 16, and 20 weeks post vaccination (Figure 5F). Taken together, our data demonstrate that PPDb-stimulated cells in each of the four unique memory CD4^+^ T cell subsets are capable of producing IFN-γ, yet there is a percentage of cells that do not produce IFN-γ. Additionally, the majority of IFN-γ-producing cells appear to arise from a population of CD4^+^ T cells exhibiting a T_EM_ phenotype.

### 3.5 Effects of PMA/ionomycin restimulation on IFN-γ production

**Figure 6:**
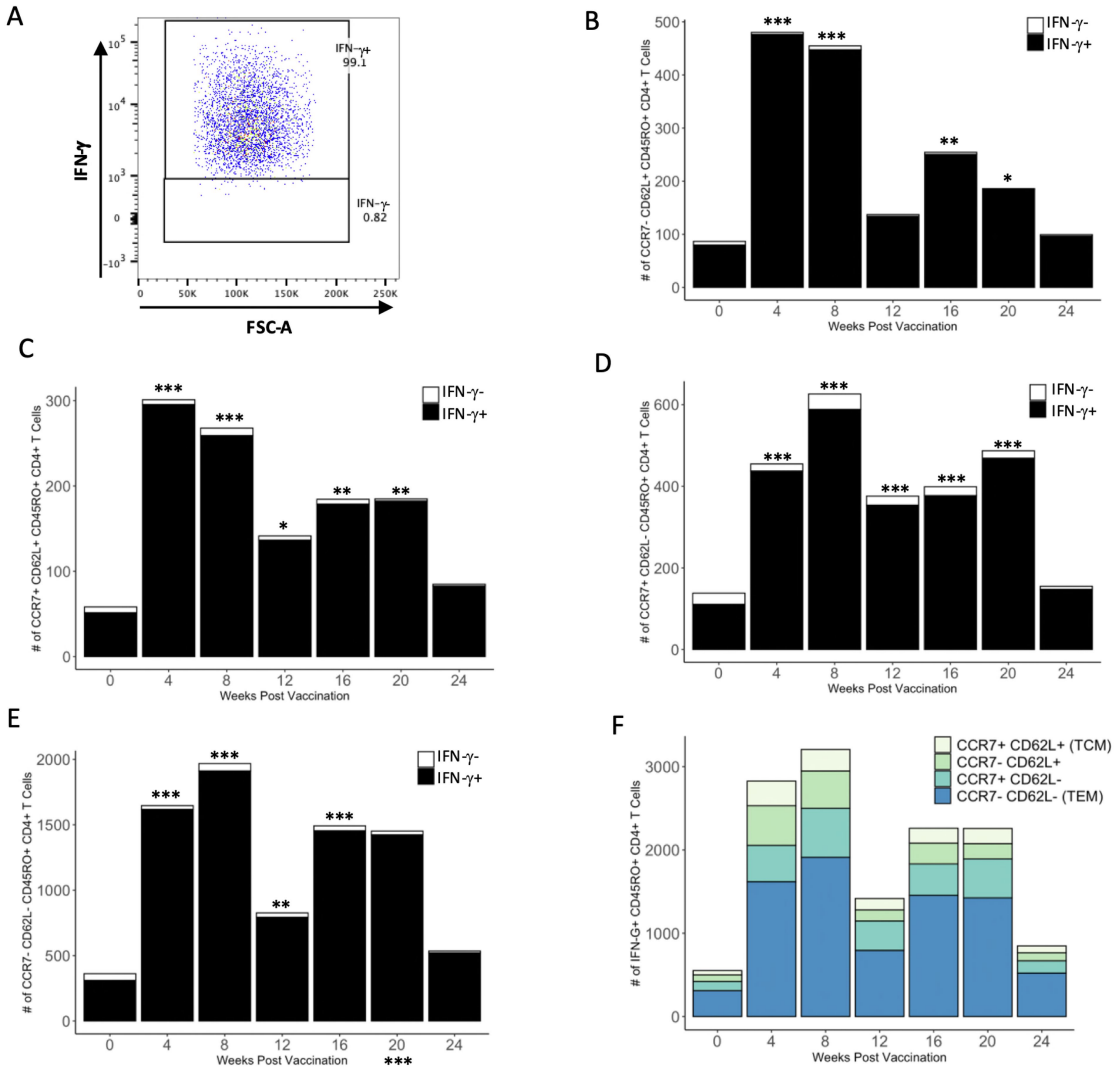
A majority of PPDb-specific CD4^+^ T cells within each CD45RO^+^ subtype have the capacity to produce IFN-γ. Representative dot plot shows gating for PPDb-specific IFN-γ^+^ and IFN-γ^−^ CD45RO^+^ CD4^+^ T cells stimulated with PMA/ionomycin. Gating is based on IFN-γ single stain control **(A)**. Number of PPDb-specific IFN-γ^+^ (black bars) and IFN-γ^−^ (white bars) CD45RO^+^ CD4^+^ T cells within the subtypes CCR7^−^ CD62L^+^ **(B)**, CCR7^+^ CD62L^+^ T_CM_ **(C)**, CCR7^+^ CD62L^−^ **(D)**, and CCR7^−^ CD62L^−^ T_EM_ **(E)**. The count of IFN-γ^+^ and IFN-γ^−^ cells represents the total number of unique CD45RO^+^ subset at each time point post vaccination **(B-E)**. Counts of IFN-γ^+^ cells within each subset are shown using a standard axis, with the total count of all four subsets being equivalent to the overall number of PPDb-specific IFN-γ^+^ CD45RO^+^ CD4^+^ T cells at each time point post vaccination when stimulated with PMA/ionomycin **(F)**. *p ≤ 0.05; **p ≤ 0.01; ***p ≤ 0.001.

To determine if the number of antigen-specific IFN-γ^+^ CD4^+^ memory T cells could be enhanced, cells were restimulated with PMA/ionomycin for the final 16 h of *in vitro* incubation with PPDb antigen. Restimulation with PMA/ionomycin caused most of the cells within each memory subset to produce IFN-γ, and very few cells in any subset remained IFN-γ^−^ (Figure 6A). Restimulation resulted in a significant increase in the number of IFN-γ^+^ cells within each memory subset at most time points (Figures 6B–E). The number of IFN-γ^+^ T_EM_ CD4^+^ T cells was significantly greater than all other subsets between 4 and 20 weeks after vaccination (Figure 6F). Collectively, our data demonstrates that the vast majority of CD4^+^ T cells within all PPDb-specific memory subsets have the functional capacity to produce IFN-γ.

## 4 Discussion

It has been well established that antigen-specific memory T cells allow for the rapid induction of a protective cell-mediated immune response against infectious pathogens (20). Effective vaccination strategies result in generation of long-lived memory T cells, which proliferate and provide effector functions upon recognition of pathogen-specific antigens. Understanding the magnitude and kinetics of the memory response induced following vaccination or infection is necessary to characterize the nature and duration of immunity.

Although there is currently no approved bTB vaccine, BCG continues to be the vaccine candidate with the most potential for adaptation for use in domestic livestock. While *M. bovis* antigen-specific CD4^+^ T cells can be expanded *in vitro* from PBMC isolated from BCG vaccinated cattle (7), investigation into the memory subsets and concurrent assessment of effector functions (i.e., proliferation and cytokine production) of memory CD4^+^ T cells has not been reported to our knowledge. Therefore, in the study presented here, we further characterized CD4^+^ T cell memory responses induced following BCG vaccination in cattle. Using proliferation as a measure of antigen specificity, we identified four PPDb-specific CD4^+^ T cell memory subsets in the peripheral blood of BCG vaccinated cattle based on surface expression of known memory T cell markers CD45RO, CCR7, and CD62L (9, 12, 16, 21). Our data demonstrates that within each subset, cells are capable of producing IFN-γ after PPDb stimulation alone or in response to additional stimulation.

Analysis of the PPDb-specific CD4^+^ T cell population revealed that the majority of proliferating cells are CD45RO^+^, indicating that memory T cells are the predominant population within the circulating CD4^+^ T cell response. This memory population is established early after vaccination with peak responses approximately 8 weeks post vaccination. Further phenotypic characterization of CD45RO^+^ CD4^+^ T cells indicate that the majority of the memory population is composed of CCR7^−^ CD62L^−^ cells consistent with a T_EM_ phenotype, while CCR7^+^ CD62L^+^ T_CM_ cells are much lower in number. The contrast in the relative contribution of T_EM_ and T_CM_ to the memory population may have been influenced by the assay used to characterize T cells as well as the location of PBMC collection. T_CM_ are not terminally differentiated and can simultaneously proliferate and transition to effector subtypes (14, 16). Since T_EM_ lack the capacity to proliferate rapidly (14), the proliferating, antigen-specific T_EM_ observed following 7 days of *in vitro* PPDb stimulation likely differentiated from PPDb-specific T_CM_. The capacity of PPDb-specific T_CM_ to expand and differentiate to T_EM_ under *in vitro* conditions may correlate to protection against bovine tuberculosis, though an appropriate challenge study would be necessary to test this hypothesis. *In vivo*, circulating T_EM_ are confined to the peripheral blood under homeostatic conditions, whereas T_CM_ recirculate through secondary lymphoid organs in addition to peripheral blood (13). Therefore, a fraction of T_CM_ were effectively inaccessible for collection in peripheral blood, resulting in an inherently higher prevalence of T_EM_ in the blood samples collected for analysis.

In addition to the classical T_CM_ and T_EM_ memory subsets typically emphasized in the literature, we also identified populations of PPDb-specific CD45RO^+^ CD4^+^ T cells that expressed either CCR7 or CD62L independently. These CCR7 and CD62L single positive CD45RO^+^ CD4^+^ T cells constituted a significant portion of the memory population in our study and were more prevalent than CD4^+^ T_CM_. CD62L and CCR7 expression on lymphocytes together mediate rolling adhesion and attachment to HEVs for lymph node entry. This extravasation of lymphocytes through HEVs is a step-wise process, and cannot occur without either initiation of rolling adhesion by adhesion molecules or firm attachment regulated by chemokine receptors (15). However, CD4^+^ T cells that express CCR7 but not CD62L have been identified *ex vivo* in the peripheral blood, spleen, and lymph nodes of mice, indicating that these surface molecules are not strictly co-regulated and can be expressed on the surface of CD4^+^ T cells independently (22). Unpublished work from our laboratory also identified CCR7 and CD62L single positive CD45RO^+^ CD4^+^ T cells in the peripheral blood of cattle in an *in vitro* recall response assay following brucellosis vaccination [unpublished data], confirming independent expression of these surface molecules in cattle. Knowing that T_CM_ proliferate and transition to T_EM_ upon cognate antigen recognition (14, 16), CCR7 and CD62L single positive memory CD4^+^ T cells may be in a transition state between T_CM_ and T_EM_. Further characterization of the distribution of CD45RO^+^ CD4^+^ T cell subsets in peripheral blood and immune tissues of cattle *ex vivo* will allow us to determine whether CCR7 and CD62L single positive memory CD4^+^ T cells are present in cattle under homeostatic conditions, or if our observation is dependent on *in vitro* antigen stimulation.

IFN-γ production by CD4^+^ T cells is a hallmark of a Th1 immune response (9), which is known to be critical for protection against mycobacterial infections (7, 8). Previous work has shown that amount of IFN-γ produced does not directly correlate to the protection provided against *M. bovis* challenge (10), though the source of IFN-γ may be of more importance (17, 23). We assessed the IFN-γ producing potential of each memory subset characterized and found that only a portion of PPDb-specific memory CD4^+^ T cells produce IFN-γ in response to antigen stimulation. This was not a subset-dependent observation, as it occurred in T_CM_, T_EM_, and CCR7 and CD62L single positive CD4^+^ memory T cell subsets. The highest number of IFN-γ^+^ cells was found to be within the most prevalent memory subset, T_EM_, coinciding with the established role of T_EM_ as rapid cytokine producers upon cognate antigen recognition (9, 14, 21). Though by definition T_CM_ CD4^+^ T cells are not major sources of IFN-γ (14, 21), studies in humans have shown that IFN-γ production by tuberculosis antigen-specific CD4^+^ T_CM_ in peripheral blood positively correlates with recovery from tuberculosis (23). Our observation of PPDb-specific IFN-γ^+^ T_CM_ in the peripheral blood of BCG vaccinated cattle coincides with previous findings which identified these cells in the peripheral blood of *M. bovis* infected cattle using an ELISPOT assay after 14 days of PBMC culture (17). We were able to demonstrate IFN-γ production by PPDb-specific CD4^+^ T_CM_ using surface marker and intracellular staining after only 7 days of PBMC culture, suggesting the time required for IFN-γ expression in this important cell population is at least half of what was previously reported.

The presence of PPDb-specific memory CD4^+^ T cells that do not produce IFN-γ led us to ask whether these cells had the potential to produce this cytokine if given additional signals. Addition of PMA/ionomycin resulted in production of IFN-γ by a vast majority of PPDb-specific memory cells within all four subsets, indicating that these cells have the capacity to express IFN-γ given additional signals. Production of IFN-γ indicates polarization of the PPDb-specific CD45RO^+^ CD4^+^ T cells to a Th1 phenotype. Th1 CD4^+^ T cells may also produce interleukin-2 (IL-2) and tumor necrosis factor-alpha (TNF- α ), or any combination of these cytokines with or without concurrent IFN-γ production (9, 24). Even though a portion of PPDb-specific CD45RO^+^ CD4^+^ memory T cells did not produce IFN-γ without PMA/ionomycin stimulation, our data demonstrate that BCG vaccination of cattle induces antigen-specific memory cells polarized toward a Th1 phenotype that require additional signals to produce IFN-γ. These data suggest that the potential to produce IFN-γ and/or other cytokines solely based on antigen stimulation may be an under-estimate of the capabilities of memory CD4^+^ T cells. Measuring cytokines in cell culture supernatants or using additional intracellular cytokine staining to further evaluate cytokine profiles after BCG vaccination or *M. bovis* challenge may allow for identification of direct correlates of protection for bovine tuberculosis.

Our data indicate that BCG vaccination of cattle induces development of four subsets of PPDb-specific memory CD4^+^ T cells with effector phenotypes consistent with a Th1 immune response, which is known to be important for protection against *M. bovis* (7–9). T_EM_ capable of producing IFN-γ were observed in high numbers after *in vitro* antigen stimulation up to 20 weeks following BCG vaccination, indicating a strong effector memory response. T_CM_ and single positive CCR7 and CD62L memory CD4^+^ T cells were observed to produce IFN-γ and contribute to the memory response in addition to T_EM_. Though our focus in this study was on the contribution of CD4^+^ T cells to the peripheral immune response to *M. bovis* in cattle, we acknowledge that protection against bTB encompasses a much more complex, heterogenous population of immune cells. Additionally, this work utilizes *in vitro* antigen stimulation of PBMCs, which may not reflect the complete *in vivo* immune environment. However, this characterization of the CD4^+^ T cell subsets and kinetics of the memory response induced by BCG vaccination in cattle will contribute to development of a more efficacious vaccine for bovine tuberculosis.

## Data Availability

The datasets presented in this study can be found in online repositories. The names of the repository/repositories and accession number(s) can be found at: Ag Data Commons 10.15482/USDA.ADC/26871160.
